# Advanced Nanobiomaterials: Vaccines, Diagnosis and Treatment of Infectious Diseases

**DOI:** 10.3390/molecules21070867

**Published:** 2016-07-01

**Authors:** Eva Torres-Sangiao, Alina Maria Holban, Monica Cartelle Gestal

**Affiliations:** 1 Department of Microbiology and Parasitology, University Santiago de Compostela, Galicia 15782, Spain; eva.torres.sangiao@gmail.com; 2 Department of Microbiology and Immunology, Faculty of Biology, University of Bucharest, Bucharest 060101, Romania; alina_m_h@yahoo.com; 3 Department of Science and Engineering of Oxide Materials and Nanomaterials, Faculty of Applied Chemistry and Materials Science, University Politehnica of Bucharest, Bucharest 060042, Romania; 4 Department of Infectious Diseases, College of Veterinary Medicine, University of Georgia, Athens (UGA), GA 30602, USA

**Keywords:** nanoparticles, vaccines, microbiology diagnosis, biofilm, antibiotic resistance

## Abstract

The use of nanoparticles has contributed to many advances due to their important properties such as, size, shape or biocompatibility. The use of nanotechnology in medicine has great potential, especially in medical microbiology. Promising data show the possibility of shaping immune responses and fighting severe infections using synthetic materials. Different studies have suggested that the addition of synthetic nanoparticles in vaccines and immunotherapy will have a great impact on public health. On the other hand, antibiotic resistance is one of the major concerns worldwide; a recent report of the World Health Organization (WHO) states that antibiotic resistance could cause 300 million deaths by 2050. Nanomedicine offers an innovative tool for combating the high rates of resistance that we are fighting nowadays, by the development of both alternative therapeutic and prophylaxis approaches and also novel diagnosis methods. Early detection of infectious diseases is the key to a successful treatment and the new developed applications based on nanotechnology offer an increased sensibility and efficiency of the diagnosis. The aim of this review is to reveal and discuss the main advances made on the science of nanomaterials for the prevention, diagnosis and treatment of infectious diseases. Highlighting innovative approaches utilized to: (i) increasing the efficiency of vaccines; (ii) obtaining shuttle systems that require lower antibiotic concentrations; (iii) developing coating devices that inhibit microbial colonization and biofilm formation.

## 1. Introduction

In 1959, Richard Feynman described a process that allows one to individually manipulate atoms and molecules throughout high precision instruments. This system could be applied to design and build systems at nanoscale level, atom by atom [1,2] and its applications in many areas of wide interest such as health, industry, pharmacy, etc., seem to be unlimited. In 1981 the engineer Eric Drexler, inspired by Feynman’s speech, published the article entitled “Molecular engineering: An approach to the development of the general capabilities for molecular manipulation” in which he described more in detail what Feynman have previously described [3]. The term “nanotechnology” was first applied by Drexler in 1986 [4] and it has been used for this area of expertise since.

Nanotechnology refers to the area of the knowledge that designs and produces structures, devices and systems by manipulating atoms and molecules at the nanoscale level [5]. Nanoparticles are microscopic particles smaller than 100 nanometers [6]. Due to their small size, nanoparticles have unusual properties which make their use in nanomedicine advantageous [7]. Nowadays most nanoparticles are obtained from transition metals, silicon, carbon and metal oxides.

Nanobiotechnology is the area of nanotechnology focused on the biological field. Nanoparticles utilized in biology are grouped into three categories: organic, inorganic and mixed (organic/inorganic) [8]. In recent years, many nanoparticles have been developed for diverse applications in medicine, including infectious diseases. The development of nanoparticles in this area has been beneficial due to their selective antimicrobial effect with low toxicity against the host and their ability to place their action on specific targets. 

Shuttle systems are commonly used for the delivery and stabilization of bioactive drugs and antimicrobial molecules, ensuring not just their specificity but also controlled release [8]. Coating medical devices is of a great advantage in the infectious disease field, e.g., nanomodified surfaces and devices proved to be very efficient to reduce microbial attachment and biofilm formation [9,10].

The use of nanomaterials as biosensors has currently a vast impact and a fast development in the usage of smart nanobiomaterials. Biosensors are accurate and offer a cost effective approach for the detection of pathogenic infectious agents in natural environment, food but also clinical specimens [11]. Nanondiagnostics was first introduced by Mirkin et al. [12] in 1996 where the authors published the use of gold (Au)-nanoparticles to allow anthrax detection [13]. 

The aim of this review is to highlight and discuss the recent progress and applications of nanotechnology in the medical field (nanomedicine) focusing on the prevention, diagnosis and treatment of infectious diseases.

## 2. Nanoparticles and Vaccines

Traditional vaccines have been developed using live attenuated organisms (cellular vaccines) or inactivated toxins or proteins (acellular vaccines). Recently, the development of synthetic peptide-based vaccines has shown many advantages compared with traditional vaccines, such as better safety and/or conservation. However, the peptide-based vaccines generate a weaker immune response, and the inclusion of adjuvants and/or the use of vaccine delivery systems is highly needed [14]. Antibacterial vaccines, both cellular and acellular, are still considered the most cost effective intervention against bacterial infections. Implementation of vaccine schedules has decreased worldwide the morbidity and mortality caused by infectious diseases such as diphtheria, pneumococcal and pertussis diseases. However, the treatment and prevention of other common bacterial infections, including but not limited to *Staphylococcus aureus*, *Helicobacter pylori, Shigella* spp. or *Escherichia coli* is still not possible [15].

Nanoparticles have several applications in nanobiomedicine, especially in the field of vaccine production where they can be applied as efficient delivery systems. Their particular nature increases cross-presentation of the peptide [16] and it also plays an important role in the activity of antigen presentation cells (APC) [16,17]. The main application of synthetic nanoparticles in immune engineering relies on the modulation of APC, by encapsulating or releasing molecules that promote dendritic cell activation, triggering particle-specific immune recognition and thus, antigen processing. Nanoparticles can further act as co-adjuvants, stimulating the proper pro- or anti-immunity pathways. This immuno-stimulation can be achieved by encapsulation of various compounds and/or according to their structure or composition [17]. In addition, hypersensitivity produced by the nanoparticles used can be ameliorated by slowing the rate of infusion of the delivery nanovaccine system, by modulating their shape and size, or by patient premedication [18].

Nanomaterials may have intrinsic immunomodulatory functions, acting as adjuvants or immune potentiators [17]. According to the nanomaterial composition [19], the vaccine-associated nanoparticles [20] could be classified in different types (Table 1).

(i) Polymers, divided in turn into nanoparticles containing synthetic polymers, such as poly(d,l-lactic-co-glycolic acid)(PLGA) [21], polyethylene glycol (PGE) [22] or polyester bio-beads [23], and natural polymers based on polysaccharides such as alginate [24], inulin [25] or chitosan [26]. Synthetic and natural polymers have been used to synthesize hydrogel nanoparticles, which have favorable properties including but not limited to flexible mesh size, large surface area for multivalent conjugation, high water content, and high antigen loading capacity [27];
(ii) Liposomes, which are biodegradable and non-toxic phospholipids. They encapsulate antigens and incorporate viral envelope glycoproteins to form virosomes. The combination of a modified cationic liposome and a cationic polymer (such as protamine)-condensed DNA is called liposome-polycation-DNA nanoparticles (LPD). They are commonly used as adjuvant delivery system in DNA vaccine studies [28];
(iii) Nanosized emulsions are those nanoparticles that can exist as oil-in-water or water-in-oil form. Emulsions can carry antigens inside their core to increase the efficiency of vaccine delivery or they can also be simply mixed with antigen [20];
(iv) Inorganic nanoparticles are non-biodegradable, they have rigid structure and controllable synthesis. Silica-based nanoparticles (SiNPs) offer the advantage of biocompatibility and have excellent properties as nanocarriers. SiNPs particles such as mesoporous silica nanoparticles (MSNs) could potentially become high-efficient, controlled-release nanocarriers in future vaccine formulations [20].
(v) Immuno-stimulating complexes (ISCOM). They are composed of supra-molecular structures of the adjuvant Quil A and immunizing peptides, which allows selective incorporation of viral envelope proteins by hydrophobic interaction [29].
(vi) Virus-like particles (VLP) are optimized for interaction with the immune system, avoiding the infectious components. They can induce potent immune responses, even in the absence of adjuvant [30]. VLP based vaccines have been the first nanoparticle class to reach market [31], found for example under the following Engerix^®^, RECOMBIVAX^®^HB against to HBV [32].
(vii) Self-assembling systems emerged as a consequence of an attempt to drive higher levels of protein, and consequently better immunological properties. A variety of natural proteins can be self-assembled into nanoparticles, conferring highly symmetric, stable and organized structure [32].

## 3. Microbial Detection by Using Nanoparticles

The emergence of infections together with the fast evolution of drug-resistant bacteria (superbugs), are triggering the increased ineffectiveness of actual therapies used to treat infectious diseases [94]. Clinical microbiology laboratories still use the conventional phenotypic methods for the identification of bacteria and novel mechanisms of resistance. Nowadays laboratories are supported by molecular biology techniques, such as those based on 16S rRNA sequencing, but also various advanced physico-chemical analysis. Matrix-assisted laser desorption/ionization time-of-flight mass spectrometry (MALDI-TOF MS) is becoming a reliable method for microbial identification in several hospitals, due to their speed, accuracy and cost effectiveness [95]. For the design of an optimal diagnosis method, some parameters should be considered: this would be a cost-effective, portable, and point of source-detection system which would be also highly reliable, sensitive, and accurate [96]. The desirable method also should be able to detect multiple pathogens in one single run.

A number of nanotechnology-based materials have been studied with the purpose of controlling and preventing infectious diseases [97]. The physical and chemical properties of nanoparticles allow accurate, rapid, sensitive, and cost-efficient diagnostics [94]. Antibody-based diagnoses such as those utilizing Fluorescent Silica Nanoparticles (FSNPs) have been developed in order to detect *Mycobacterium* *tuberculosis* complex (MTB) within 4 h [98]. Incorporating europium [Eu(III)] polymeric nanoparticles have been successfully for the detection of anthrax antibodies by using fluorescence enzyme linked immunosorbent assay (ELISA) [94]. A combination of positive di-electrophoresis and aptamer-FSNPs label has been developed as a rapid and sensitive method for detection of *S. aureus* [99]. Wang and Kang [100] have developed recently, a method for detection of *Salmonella typhimurium* based on a single-stranded DNA aptamers along with silica fluorescence nanoparticles.

Liposomes can recognize target toxins, and therefore they can be used for toxin detection. Liposomes labelled with fluorescent markers (such as rhodamine dyes) can be incorporated into sandwich fluoro-immunoassay on antibody-coated microtiter plates in order to detect toxins [101]. Ahn-Yoon et al [102], had used this method to detect cholera toxin within a limit of detection of 10 fg/mL and in only 20 min [102]. A similar assay was developed to detect botulinum toxin (BT) on a nitrocellulose membrane strip by using tri-sialo-ganglioside GT1b-liposomes, which is a receptor for BT [103].

Quantum dots (QDs) are special nanocrystalline semiconductors [94] composed of materials such as ZnS, ZnSe, CdS, CdSe, CdTe and InP, among others [104]. QDs show strong resistance to photobleaching and chemical degradation, as well as significant photostability and high quantum yield [105]. These characteristics make them suitable for sensitive image acquisition and signal amplification in real time [94]. The applications of QDs in nanobiomedicine are diverse, varying from fluorescent probes, biosensors to therapeutics agents [104]. Numerous methods have been developed for creating hydrophilic QDs [106]. Among QDs’ bioapplications, it is important to highlight, multiplex detection of analytes based in single molecule detection. QD-based nanosensors are an example of a highly sensitive, extremely low cost-per-sample technique, that ensures short analysis time and it has the potential to be applied for rapid detection of viral and bacterial proteins, with enhanced sensitivity and specificity over conventional organic fluorophores [104].

In 2010, Zhang and Hu [107] developed a multiplex assay for the detection of HIV-1 and HIV-2. This single-QD-based nano-sensor showed an extremely low sample consumption, high sensitivity and short analysis time. These results have shown the many advantages of this method to be applied for rapid point-of-care testing, gene expression studies, high-throughput screening, and clinical diagnostics. Six years later, Zhang et al. [108] designed an efficient immunosensor-based technique for screening and isolating *Salmonella* sp. with a detection limit of 10 cell/mL. The aforementioned fluorescent nanobioprobes made on a specially designed cellulose-based swab could be applied in a large number of samples related to public health surveillance to visually detect and directly isolate pathogens in situ.

In 2007, Klostranec et al. [109] reported the use of QDs with microfluidics for the obtention of bio-imaging signals, improving the high sensitivity for their use in diagnosis. QD-antibody conjugates has also been successfully used in fluoro-immunoassays for the detection of staphylococcal enterotoxin B [110], syncytial respiratory virus [111] or hepatovirus, and HVB, HCV, and HIV viruses [112]. Ebrahim et al. [113] have been able to synthesize CdTe-QDs conjugated with concanavalin A for the detection of lipopolysaccharide (LPS) produced by *Serratia marcescens* with a detection range from 10 to 10^6^ colony forming units/mL (CFU/mL) at pH 7.

Detection systems based on noble metal nanoparticles (Table 2), particularly Au and Ag, have been widely studied due to their unique optical and physicochemical properties [114] and they are known as surface plasmon resonance (SPR) [94]. Their nano-size scale and their optical/physicochemical properties have been used for selective and specific identification of DNA/RNA sequences, proteins, or small analytes associated with the presence of infection and various pathogens. Their detection relies on colorimetric assays, fluorescence, mass spectrometry, electrochemical, and scattering approaches [95]. In 2005, Duan et al. [115], reported the usage of immune-gold silver staining with Au-nanoparticles as a very sensitive method for the detection of single molecules and its application for the detection of HCV and HBV.

**Magnetic nanoparticles** (MNPs) have nanoscale sizes, which mimic the size of molecules in nature, and they harbor favorable characteristics for their use in nano-biomedicine, such as imaging and therapy [105]. Surface modification of MNPs with recognition moieties, for instance, antibodies, antibiotics, and carbohydrate, enables their use for bacterial detection.

These recognition moieties help to detect the bacteria selectively and at low concentrations [94,116]. The super-paramagnetic property provides MNPS with a promising and sophisticated platform for in vivo detection techniques and have the potential to make microbiological diagnostics become much easier and more worthy [116,117] (Table 2). MNPs can be classified as metal, alloys or oxides, and are generally based on elements such as Fe, Co, Ni, or Mn, among others [105]. Iron oxide nanoparticles (IONPs) are the most studied and are composed of magnetite (Fe_3_O_4_) or maghemite (γ-Fe_2_O_3_) nanocrystallites. IONP-biosensors have been developed for the detection of HSV-1 and adenoviruses enabling to detect five viral particles in 10 μL serum samples without previous PCR amplification steps [118]. Using IONPs functionalized with IgG [119] and vancomycin [120] have allowed to push the limit of detection to 10^4^ CFU/mL bacterial cells by using nano-MALDI platforms [121]. Nanodiagnostic systems will allow microbiologists to perform molecular tests faster and with higher sensitivity. These methods also increase flexibility at reduced costs [122]. However the majority of these new nanoplatforms still need further evaluation and validation with clinical samples before they can be fully translated into clinical diagnosis.

## 4. Nanoparticles for Fighting Superbugs

Drug resistance is of a great concern for public health. The use of high dose antibiotic treatments often generates high rates of toxicity and the development of new resistance. In addition, the costs of treatments increase while there is a major number of treatment failures and high spectrum therapies associated with an increase in the number of hospitalization days. Due to the lack of new alternatives for the treatment of infectious diseases, several classes of antimicrobial nanoparticles and nanocarriers for antibiotic delivery have been studied, as well as their effectiveness for the treatment of infectious diseases, including antibiotic resistant bacteria [150].

**Figure 1:**
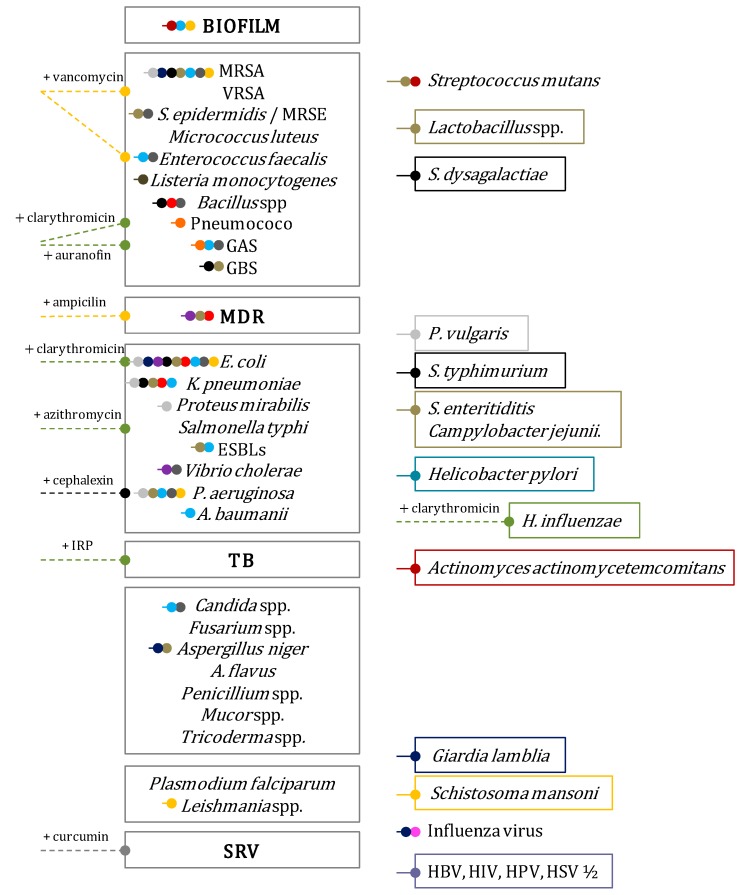

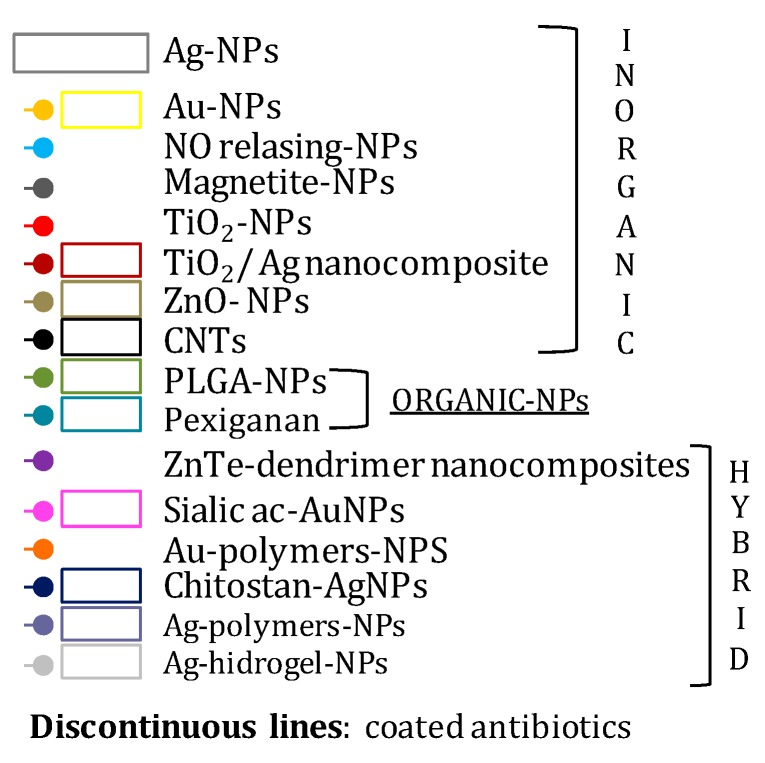
Representative uses of the main nanoparticles developed for the treatment of infectious diseases. *Abbreviations*: **CNTs**: Carbon nanotubes; **ESBLs**: expanded spectrum beta lactamases; **GAS**: *Streptococcus* group A (*S. pyogenes*); **GBS**: Streptococcus group B (*S. agalactia*e); **HPV**: human papiloma virus; **HSV**: herpes simplex virus; **IRP**: Isoniazide-rifampicin-paramycin; **MDR**: multi-drug-resistance; **MRSA**: methicillin resistance *S. aureus*; **MRSE**: methicillin resistant *S. epidermidis*; **NPs**: nanoparticle; **PLGA**: poly(d,l-lactic-co-glycolic acid); **SRV**: syncital respiratory virus; **TB**: tuberculosis (*Mycobacterium tuberculosis*); **VRSA**: vancomycin resistant *S. aureus.*

Nanoparticles provide a versatile platform for the design of materials with antimicrobial properties. Their unique nanoscale as well as physical and chemical properties provide multiple attributes that facilitate the development of unique antimicrobial strategies; hence, they are emerging as weapons in our antimicrobial arsenal. These nano-antimicrobial materials can be synthesized by variety of different methods influencing subsequent antimicrobial effect [151]. They could be divided into inorganic, organic and hybrid nanoparticles. The most advantageous are inorganic nanomaterials, such as Ag and Au, alone or combined with various organic polymers (Figure 1).

The antimicrobial mechanism of the action of nanoparticles is not fully known. Nevertheless, the antimicrobial actions include destruction of cell membranes, blockage of enzyme pathways, alterations of microbial cell wall, and nucleic materials pathway. The applicability of nanoparticles as therapeutic agents includes a wide range of action, varying from broad spectrum antimicrobial agents, sterilization and wound healing agents, to sustained inhibitors of intracellular pathogens [152]. The most of the tested nanoparticles are highly efficient against *Staphylococcus aureus* and *Escherichia coli*, and according to properties they have been even used to treat tuberculosis (TB) [36] (Figure 1).

The antibacterial activity of Ag-nanoparticles is well established, although they face certain shortcomings due to toxicity to mammalian cells and limited penetration in biofilm matrices [153]. Recent studies [154] have been focused on countering these issues for example by developing Ag ring-coated super-paramagnetic IONPs (SPIONS) with ligand gaps. This has demonstrated high antimicrobial activity and remarkable compatibility with healthy host cells, which further exhibited enhanced activity against biofilm infections due to deeper penetration under an external magnetic field [155]. Others inorganic nanomaterials such as Au, Cu, Ni, Ti, Zn, graphene-based photo-thermal, as well as their coupled derivatives, are potential candidate for enhancing or restoring the already existing antibiotics or new substances to combat the multi-drug-resistance (MDR) problem (Figure 1).

The nanoparticles are indeed potential broad spectrum antibiotics because they can inhibit a wide range of MDR bacteria which have defied most of antibiotic treatments [152]. For example, CuO-nanoparticles exert their antibacterial activity by membrane disruption and ROS production [156], showing an antibacterial efficacy alike to Ag or ZnO. On the other hand, ZnO–nanoparticles, which are more effective, affect bacterial cell along two pathways: (1) by binding to membranes, disrupting their potential and integrity, and (2) by inducting ROS production [151]. Hence, ZnO–nanoparticles inhibit the growth of MSSA, MRSA, MDR or pathogens such as *Streptococcus mutans*, *Lactobacillus*, *Klebsiella pneumoniae* or *E. coli* [151], including ESBL producers [157], but also prevent biofilm formation [158] (Figure 1).

Nanoshuttle systems deliver antibiotics to a precise location and release them progressively in a controlled manner (shuttle systems). These systems use nanoparticles for the delivery and controlled released of several antibiotics and natural products. Nanoparticles are free to move uninhibited into cells, increasing their efficiency. Antibiotics can be released inside the microorganism, increasing the therapeutic index and reducing the overall serum concentration. As a result, the deleterious side effects decrease [159]. An additional advantage is the decrease risk of creating resistance in other commensal microorganisms [160]. Most applications are focusing on the treatment of osteomyelitis [161], skin or wound [162] or *S. aureus*, *E. coli* or *Pseudomonas aeruginosa* infections [163]. The antimicrobial nanomaterials currently in use or under investigation are based on Ag, magnetite, TiO_2_ and ZnO. Nanotechnology is also making great progress in combating infections associated with medical devices (such as those related with biofilms formation), with the implementation of tailored coating systems. These systems are based on coating the surface with nanoparticles inhibiting biofilm formation. Most studies have been focused on pathogens frequently associated with nosocomial infections such as *S. aureus*, *P. aeruginosa*, *Acinetobacter baumani* and *K. pneumoniae* [160] (Figure 1). Min et al. [164] have demonstrated the applicability of coated degradable multilayer prosthesis. These coated prosthesis sequentially deliver the antibiotic and the osteo-inductive growth factor (BMP-2). This coating delivery system enables both eradication of established biofilms, as well as, a complete and rapid bone tissue repair around the implant in rats with induced osteomyelitis [164]. Their findings demonstrated the potential of this layered release strategy. Milo et al. [165] published a novel and previously unreported application of a pH-responsive polymer, in a dual-layered surface coating for urinary catheters that provides a visual early warning of *Proteus mirabilis* infection and their subsequent blockage control.

Nanoparticles keep offering promising alternatives in the design of effective next-generation therapeutics against bacterial, viral and fungal threats [155]. The perspective of developing powerful nano-antimicrobial agents with multiple-functionality will revolutionize clinical medicine and it will play a significant role in alleviating disease burden [152]. Nanoparticle-based antimicrobial agents can be used in ex vivo applications such as sterilizers for surfaces and devices, and the prospective topical applications for wound healing of nanoparticles-based systems [155] (Figure 1).

Currently, some drug delivery systems (DDS) usually named “nano-antibiotics” have been clinically-approved for human use in various infectious diseases, among them, liposomal delivery systems. Pulmaquin™ and Lipoquin™ (Grifols, S.A., Barcelona, Spain and Aradigm Corporation, Hayward, CA, USA) are inhalable liposomal dosage forms of ciprofloxacin, for the treatment of serious infectious diseases encountered in cystic fibrosis (CF) or in non-CF bronchiectasis. AX-Tobra™ (Axentis Pharma, Zurich, Swizerland) based on Fluidosomes^®^ technology and Arikace^®^ (Insmed Inc., Monmouth Junction, NJ, USA) undergoing phase III clinical trials, are respectively, an inhalable liposomal tobramycin and amikacin dosage forms, claimed for the treatment of *P. aeruginosa* pulmonary infections in cf. [166].

## 5. Conclusions

Nanobiotechnology offers multiple solutions for the prevention, diagnosis and treatment of infectious diseases. Nanoparticles can be designed to increase the activity in vaccines with low toxicity against the host. A huge number of nanoparticles can be used for the delivery and stabilization of bioactive drugs as well as antimicrobial molecules, ensuring controlled release of the drug. Due to their specificity, low dimensions, targeted delivery, controlled release properties and low cytotoxicity, nano-active systems could lead to more efficient and less invasive therapeutic outcome, contributing to the development of personalized treatment for several infectious diseases.

In the superbug era, nanotechnology is offering a new approach that allows us to fight against resistant bacteria. Nanobiomedicine offers new tools to be applied in the prevention, detection and treatment of the infectious diseases, managing to decrease the co-morbidity/mortality ratios, costs and improving lifestyle quality.

## Figures and Tables

**Table 1:** Most representative vaccine applications based on nanotechnology.

Type of Nanoparticles		Based on	Main Characteristic	Use	Representative Uses	Ref.
**Polymers**	**Synthetic**	**PLG**	Biocompatibility & biodegradability	entrap antigen for delivery (*carrier*) to certain cells and sustain Ag release according to their biodegradation rate	Toxoplasmosis	[33]
					HIV	[34]
		**PLGA**			*S. aureus*	[35]
					TB	[36]
					*Brucella abortus*	[37]
					Antrhrax	[20]
					*Plasmodium vivax*	
					HBV	
		**PGE**			Influenza Virus	[38]
					HIV	[39]
		**Polystyrene**	Can be conjugated with a variety of Ag or surface-modified with various functional groups		*P. malariae*	[40]
		**Polyester Bio-Beds**	Vaccine delivery system low cost & biocompatibility		TB	[41]
	**Natural**	**Inulin: ADVAX™**	Activator of complement alternative pathway, potent adjuvant.	adjuvant	Antrax	[42]
					*Listeria monocytogenes*	[43]
					Influenza virus	[44]
					SARS-CoV	[45]
					HBV	[25]
					HIV, JVE-WNV	[46]
		**Alginate**	Biocompatibility, biodegradability & nontoxic		*K. pneumoniae*	[47]
					*P. aeruginosa*	[48]
		**Pullulan**			Influenza virus	[49]
					Difteria	[50]
		**Chitosan**				
			Easily modified		*E. coli* O157:H7	[51]
					*P. aeruginosa*	[52]
					Influenza virus	[38,53]
					HBV	[54]
					Filariasis	[55]
					Dengue	[56]
	**Hydrogel**	**Pullulan**	Flexible mesh size Large surface area for multivalent conjugation: high capacity for Ag	hydrophilic 3D polymer network.	*S. pneumoniae*	[57]
		**Chitostan**			*C. botulinum*	[20]
					*S. pneumonie*	[58]
					Papilomavirus	[59]
					NDV	[60]
					Dengue	[61]
**Liposomes**	**LPD**	phospholipids	Biodegradable & nontoxic encapsulate Ag and form virosomes	adjuvant	*P. malarie*	[40]
					Influenza Virus (INFLEXAL^®^ V)	[62]
					HAV (Epaxal^®^)	
					HIV	[63]
	**ICMV**			adjuvant *carrier*	*P. vivax*	[40]
**Emulsions**	**Oil-in-water/water-in-oil**	**MF59™**	Mixed with Ag & transport	adjuvant	*S. aureus*	[64]
					Influenza Virus (FLUAD^®^, AFLUNOV^®^, FOCETRIA^®^, OPTAFLU^®^)	[62]
					HAV (Epaxal^®^)	
	
		**AS03/AS04**			Inluenza Virus (Pandemrix^®^, Prepandrix^®^)	[62]
					HBV (Fendrix^®^)	
					Papilomavirus (Cervarix^®^)	
		**Montanide** **™**			*S. aureus*	[65]
					*Plasmodium* spp.	[66]
					*L. amazonensis*	[67]
**Inorganic**	**AuNP**	**Au/gold**	APC cytokine production can be induced according to shape and size	Adjuvant recognition, absorption of specific biomolecules, improvement of interaction with cells & enhancement of cellular uptake	Pneumococo	[68]
					*L. monocytogenes*	[43]
					*Burkholderia mallei*	[69]
					*Yersinia pestis*	[70]
					*P. falciparum*	[71]
		**GNP**			HIV	[72]
	**CNT**	**Carbon**	Good biocompatibility Synthesized into a variety of nanotubes and mesoporous spheres multiple copies of protein and peptide Ag		*P. vivax*	[73]
	**SiNP**	**Si**	Biocompatible		*E. coli* 0111	[74]
					Influenza Virus	[75]
					HBV	[76]
	**calcium**	**Ca**	Excellent biocompatibility & non-toxic for DNA vaccines and mucosal immunity		Enterovirus 71	[77]
					NDV	[78]
					HIV	[79]
**ISCOM**		Quil A, cholesterol, phospholipids & protein Ag	Trap the Ag by apolar interactions mucosal immunity	Adjuvant	*S. aureus*	[80]
					*P. malarie*	[40]
					Chagas disease	[81]
					Tetanus	[82]
					Influenza Virus	[20]
					HSV	
					HBV	
					HIV	
**VLP**		Self-assembly biocompatible capsid protein	Evolved viral structure & delivery platform	Induce potent immune responses	Papilomavirus (Cervarix^®^, GARDA SIL^®^)	[62]
					HBV (Engerix^®^, RECOMBIVAX^®^HB)	
					HIV, Influenza Virus, Marburg, Ébola	[83]
					*E. coli*,	[32]
					*P. falciparum*	
					Norovirus, HEV (Hecolin)	[84]
					VZV	[85]
					HVC	[86]
					Enterovirus	[87]
					Chikunguya Virus	[88]
					*S. pneumoniae*	
**Self-assembling systems**	**ferritin**	Fe	Attempt to drive higher levels of protein quaternary structuring	Adjuvant	Influenza Virus, VEB, HCV, HIV	[32]
	**MVP**	Protein			HIV	[89]
	**SAPNs**	Peptides	Ability to repetitively present Ag Better biophysical & immunological properties	Strong immunogenic effect (of cellular vaccines) Purity & high specificity immune responses.	*P. malarie*	[90]
					Toxoplasmosis	[91]
					Influenza Virus	[92]
					HIV	[93]
					HCV	[85]

**Ag**: antigens; **AuNP**: Gold nanoparticles; **GNP**: gold glyco-NPs; **HAV**: Hepatitis A Virus; **HEV**: Hepatitis E Virus; **HSV**: Herpes Simple Virus; **ICMVs**: Liposomes modified with maleimide synthesized into interbilayer-crosslinked multilamellar vesicles; **ISCOM**: Immuno-stimulating complex; **JEV**: Japanese encephalitis virus; **LPD**: liposome-polycation-DNA nanoparticles; **MSNs**: mesoporous silica nanoparticles; **MVP**: vault protein; **NDV**: Newcastle disease virus; **PGE**: poly(ethylene glycol); **PLG**: poly(d,l-lactide-co-glycolide); **PLGA**: poly(d,l-lactic-coglycolic acid); SAPN: self-assembling peptide nanoparticles; **SARS-CoV**: Severe acute respiratory syndrome-associated coronavirus; **VEB**: Virus Epstein-Bar; **VZV**: Varizela Zoster Virus; **WNV**: West Nile virus.

**Table 2:** Microbiological diagnosis approach by inorganic nanoparticles.

Nanoparticle	Based on	Detection/Identification by	Detection	Limit of Detection	Ref.
**Surface-Enhanced Raman scattering Spectroscopy (SERS)**					
**AgNPs**	Label-free near infrared surface-enhanced Raman scattering (NIR-SERS)	Spectrum	MRSA, *Listeria* spp., *E. coli & P. aeruginosa*	10^3^ CFU/mL	[123]
**Au-cotted-NPs**	Surface-enhanced Raman scattering spectroscopy (SERS)		*Legionella* spp.		[124]
**Vancomycin coatted Ag-Au-NPs**			*S. epidermidis, B. megaterium*, *E. coli & Salmonella enterica*	10^2^ CFU/mL	[125]
**Surface Plasmon resonance (SPR). NanoProbes**					
**AuNPs**	Differential stabilization of Au-nanoprobes in presence of DNA targets following salt induced aggregation	Colorimeric detection from red to blue	TB	0.75 μg 2 h	[11]
	Different interaction between ssdna and dsdna at the surface of aunps, based on the aggregation of unmodified aunps		*Bacillus. anthracis*		[126]
	*Cross-linking* approach, where the target DNA acts as a linker between two different Au nanoprobes; based on aunp aggregation	Colorimetric detection from red to blue	MRSA	66 pg/μL (<10^5^ CFU/mL)	[11]
	Interaction aunps–dsdna & the addition of thiolated probes specific to the *inva* gene in the *Salmonella* genomic DNA aggregates aunps	Colorimetric detection from red to violet	*S. enterica*	37 fM	[127]
	The ability of ssdna oligo-targeters to stabilize the colloidal aunps preventing their salt-induced aggregation.	Colorimetric detection	*Acinetobacter baumani*	0.8125 ng/μL	[128]
	*Non-cross-linking* method results from the differential aggregation profiles of Au-nanoprobes induced by increased ionic strength in the presence or absence of the specific target sequence	Colorimetric detection (SPR band: 525–650 nm)	MTBC and *Plasmodium*		[129]
			MDRTB		[130]
	Multichannel fluorescence sensor	Ratiometric response according to three-color RGB output	BIOFILMS: *Amycolatopsis azurea*, *B. licheniformic*, *B. megaterium*, *E. coli*, *P. aeruginosa*		[131]
	Fast lateral flow immunoassay (FLFI) approach combined with rapid “one step” lysis	Colorimetric detection	*E. coli*	5 × 10^4^ CFU/mL 25 min	[132]
**Aptamer-conjugated-AuNPs**	aptamer–DNA duplex formed by the hybridization reaction between the capture probe and the aptamer probe, which induces a clear enzymatic catalysis of the oxidation of methionine by hydrogen peroxide	Biosensor	*C. difficile*	1 nM	[133]
	glassy carbon electrode modified with graphene oxide and AuNPs	Electrochemical impedance spectrum	*Salmonella*	3 CFU/mL	[134]
	cell-based SELEX (Systematic Evolution of Ligands by Exponential Enrichment), and dissociation constants and binding specificity	Resonance light-scattering–detection system	*S. aureus*	10 CFU/mL 1.5 h	[135]
**AuNPs paper-paper**	*Non-cross-linking* assay wax-printed microplate paper platform	Colorimetric detection from red to blue	TB	30 μg/mL 2 h	[136]
**Microarrays**					
**AuNPs**	Multiple capture and intermediate oligos to detect a target in multiple regions	Silver signal by scanomatric detection	Influenza Virus H5N1	<10^5^ copies of transcribed RNA; 2.5 h	[137]
**Ag-Au core shell NPs**	Nanoparticle-based microarrays using a photodiode sensor	SEM images	HPV	0.05 pmol/μL	[138]
**Magnetic nps**					
**AuMNPs**	*Non cross-linking* aggregation phenomenon: specific interaction between *mec*a gene with the gold	Colorimetric detection (λ = 530 nm)	MRSA		[139]
	Electrochemical geno-sensing assay onto the latex microspheres	AuNPs signal	*Vibrio cholerae*	2 CFU/mL	[140]
**Fept@Vanco**	Trapping gram-positive bacteria, based on interaction between the heptapeptide backbone of vancomycin and the d-alanyl-d-alanine dipeptide from the cell wall	MALDI-TOF	*Staphylococcus* spp., VRE & *E. coli*	100 CFU/mL	[116]
**Immunoassay**					
**Au-NPs**	AuNPs bound to anti-human IgG	Colorimetric immunoassay	Influenza Virus	10 pg/mL	[141]
	FLFI combined with ELISA		*E. coli* 0157:H7	10^3^ CFU/mL	[142]
	Plasmonic ELISA (ELISA with enzyme-mediated SPR of AuNPs)		SIFILIS	0.98pg/mL	[143]
**Ag-NPs**	SERS enzyme-catalyzed immunoassay	RAMAN Intensity	SRV	0.05 pg/mL	[144]
	ELISA, antigen-antibody immunoreaction	Chemiluminescence	*Salmonella* spp.	50–100 CFU/mL	[145]
**Eu(III)-NPs**		Fluorescence signal	HIV-1 p24	<0.1 pg/mL	[146]
**AgNPs-G**	gold electrode coated with AuNPs-G, whose is modified with H7-monoclonal antibodies	Electrochemical immunosensor	Aviar Influenza Virus H7	1.6 pg/mL	[147]
**FSNPs**	highly fluorescent bioconjugated nanoparticles probe	Fluorescence signal	*L. monocitogenes*	50 CFU/mL	[148]
**Fluorescence**					
**Si-MNPs**	high specificity for dsDNA and bright fluorescence upon intercalation into dsDNA.	Nucleic-acid dye SYBR Green I signal (Intensity)	*S. aureus*	50 CFU/mL	[149]

**AgNPs-G**: silver nanoparticle-graphene; **HPV**: Humam Papiloma Virus; **FLFI**: fast lateral flow immunoassay; **MDRTB**: Multi Drug resistance TB; **MRSA**: Methicillin resistan *S. aureus;* **MTB**: multidrug resistant TB; **MTBC:** *Micobacteriun tuberculosis complex;* **SERS**: Surface-enhanced Raman scattering spectroscopy; **SPR**: Surface Plasmon resonance; **SRV**: Syncytial Respiratory Virus; **TB**: Tuberculosis; **VRE**: Vancomycyn Resistant *Enterococcus* spp.

## Abbreviations

ACP	Antigen Presenting Cells
Ag	Antigen
BT	Botulinum toxin
CF	Cystic fibrosis
CFU	Colony Forming Unit
ELISA	Enzyme Linked Immunosorbent Assay
FSNP	Fluorescent Silica Nanoparticles
HBV	Hepatitis B Virus
HCV	Hepatitis C Virus
HIV	Human Immunodeficiency Virus
Ig	Immunoglobulin
IONP	Iron oxide nanoparticles
MALDI-TOF MS	Matrix Assisted Laser Desorption Ionization—Time of Flight Mass Spectrometry
MNP	Magnetic nanoparticles
QD	Quantum dots
ROS	Reactive Oxygen Species
SiNP	Silica-based nanoparticles
VLP	Virus-like particles
WHO	Word Health Organization
